# A Lack of Immune System Genes Causes Loss in High Frequency Hearing but Does Not Disrupt Cochlear Synapse Maturation in Mice

**DOI:** 10.1371/journal.pone.0094549

**Published:** 2014-05-07

**Authors:** Melissa A. Calton, Dasom Lee, Srividya Sundaresan, Diana Mendus, Rose Leu, Felix Wangsawihardja, Kenneth R. Johnson, Mirna Mustapha

**Affiliations:** 1 Department of Otolaryngology-Head & Neck Surgery, Stanford University, Stanford, California, United States of America; 2 The Jackson Laboratory, Bar Harbor, Maine, United States of America; University of Washington, Institute for Stem Cells and Regenerative Medicine, United States of America

## Abstract

Early cochlear development is marked by an exuberant outgrowth of neurites that innervate multiple targets. The establishment of mature cochlear neural circuits is, however, dependent on the pruning of inappropriate axons and synaptic connections. Such refinement also occurs in the central nervous system (CNS), and recently, genes ordinarily associated with immune and inflammatory processes have been shown to play roles in synaptic pruning in the brain. These molecules include the major histocompatibility complex class I (MHCI) genes, *H2-K^b^* and *H2-D^b^*, and the complement cascade gene, *C1qa*. Since the mechanisms involved in synaptic refinement in the cochlea are not well understood, we investigated whether these immune system genes may be involved in this process and whether they are required for normal hearing function. Here we report that these genes are not necessary for normal synapse formation and refinement in the mouse cochlea. We further demonstrate that *C1qa* expression is not necessary for normal hearing in mice but the lack of expression of *H2-K^b^* and *H2-D^b^* causes hearing impairment. These data underscore the importance of the highly polymorphic family of MHCI genes in hearing in mice and also suggest that factors and mechanisms regulating synaptic refinement in the cochlea may be distinct from those in the CNS.

## Introduction

In the sensory epithelium of organ of Corti, information about the timing, frequency and intensity of sounds is transmitted from and to the hair cells via highly organized synapses. While early brain development is characterized by an overabundant outgrowth of axons and dendrites that make connections with numerous targets, the formation of mature neural circuits requires activity-dependent axonal pruning and elimination of unnecessary axons and synapses. Similar synaptic refinement occurs in the cochlea as the murine organ of Corti matures into a fully functioning organ during the first two postnatal weeks.

The mature organ of Corti contains two types of sensory cells known as inner (IHC) and outer hair cells (OHCs) that are innervated by the spiral ganglion neurons (SGNs). The IHCs are considered the primary sensory cells responsible for transmitting neural signals via the afferent nerve, whereas the OHCs are responsible for signal amplification by virtue of their motor activity, which modulates IHC function [1]–[6]. Adult IHCs are innervated by 90% to 95% of type I SGNs, or SGI. About 15 to 20 unbranched myelinated SGIs connect with a single IHC while each neuron receives input from only one IHC [7], [8]. The remaining 5 to 10% of SGNs are type II unmyelinated neurons, SGII, that project toward OHC and spiral toward the base to contact multiple OHC [8]–[12]. At birth, SGI project branched neurites that innervate several targets including IHCs and OHCs. OHCs are mainly innervated with afferent fibers at birth. The mature connectivity in the organ of Corti is established by pruning of inappropriate connections including the retraction of SGI branches and the death of excess SGII. Several growth factors and guidance cues such as neurotrophins and ephrins are involved in spiral ganglion outgrowth, attraction, and repulsion. Although these factors have been identified, the specific molecular cues necessary for afferent/efferent cochlear fiber refinement remain poorly understood and warrant further investigation.

Recently, genes ordinarily associated with immune and inflammatory processes have been shown to have roles in synaptic refinement, including the MHCI (major histocompatibility complex class 1) and *C1qa*, a member of the classical complement cascade. MHCI is a large and highly polymorphic family of over 50 genes. Down-regulation of MHCI was shown to prevent retinal refinement in a screen to identify factors involved in synapse pruning in the visual system, indicating a role for this protein in these processes [13]. Beta-2 microglobulin (*β2M*) gene expression is necessary for MHCI proteins to display on the cell surface and mice lacking the *β2M* gene retain immature patterns of innervation in the brain [13]. In the retina and cerebral Purkinje cells, MHCI proteins have been found at excitatory and inhibitory synapses [14], [15]. Furthermore, a knockdown of the mouse MHCI genes *H2-K^b^* and *H2-D^b^* resulted in a dramatic increase of synapse number in the cerebellum, indicating a role in synaptic plasticity [14].

C1qa is a soluble, secreted protein, and is the initiating protein in the classical complement cascade that binds to foreign material marked for phagocytosis and clearance during an immune response. In the postnatal brain, C1qa is expressed by astrocyte-stimulated neurons [16]. Mice deficient in *C1qa* exhibit large sustained defects in CNS synapse elimination in the retina and in the cerebral cortex [16], [17]. The function of *C1qa* in the CNS seems to be similar to its role in the immune systems, clearing cellular material marked for destruction [16]. Furthermore, a reactivation of *C1qa* expression in the brain has been associated with neuronal degenerative diseases. C1qa protein increases with aging in mouse and human brains in regions associated with neurodegenerative disease, whereas *C1qa*-deficient mice have less cognitive and memory loss [18].

A recent study found that MHCI genes *H2-K^b^* and *H2-D^b^* as well as *C1qa* are upregulated during the period of cochlear functional development [19]. In this study, we examined whether these genes play a role in synaptic maturation of the neonatal mouse cochlea.

## Materials and Methods

### Mice

All experiments were completed with national animal care guidelines and were approved by the Stanford University Administrative Panel on Laboratory Animal Care; APLAC 23001.

*H2-K^b^D^b−/−^* double knockout mice on a C57BL/6 genetic background were obtained from Dr. Carla Shatz (Stanford) [15], [20] and maintained as a homozygous breeding colony because both the targeted loci are now on the same chromosome [21]. C57BL/6 controls were purchased from The Jackson Laboratory (Bar Harbor, Maine) and were bred and maintained in our facility. *C1qa* knockout mice (hereafter referred to as *C1qa^−/−^*) on C57BL/6 background were generously provided by Drs. Ben Barres and Marina Botto [22] and littermates were used as controls. Congenic *D2.B6-C1qa^+/+^*, *D2.B6-C1qa^+/−^*, and *D2.B6-C1qa^−/−^* mice were established by an intercross of *DBA/2J-C1qa^+/+^* and *D2.B6-C1qa^−/−^* F1 hybrids resulting in the F2 progeny with *C1qa^+/+^*, *C1qa^+/−^*, and *C1qa^−/−^* genotypes all on a DBA/2J background [23]. The study was carried out in strict accordance with recommendations in the Guide for the Care and Use of Laboratory Animals of the National Institutes of Health. All experiments were conducted under guidelines of the Stanford University Administrative Panel on Laboratory Animal Care. All hearing tests were performed under anesthesia, and all efforts were made to minimize suffering.

### Auditory Brainstem Responses (ABR)

ABR recordings were performed at the Auditory Core for Department of Otolaryngology, Stanford University. The presentation of stimuli, ABR acquisition, equipment control and data management were coordinated using the computerized Intelligent Hearing System (IHS; Miami, FL). Three needle electrodes were placed under the skin and animal body temperature was maintained at 37°C. Clicks and 8- 16- and 32-kHz tone-bursts were channeled through plastic tubes into the animal’s ear canals. Sound levels were incremented in 5 dB steps from 10–20 dB below threshold to 80 dB (for 8 and 16 kHz) or 100 dB (for 32 kHz). Threshold for ABR was defined as the lowest stimulus level at which repetitive waves I and V could be identified in the response waveform. The amplitude analysis was done by peak-to-peak measurement of the ABR waveform and latency was calculated as time delay from the onset of the stimulus (0 ms) until the occurrence of the ABR response peak. ABR waveform amplitude and latency analysis was performed at the 16 kHz frequency at 40 dB.

### Distortion-Product Otoacoustic Emission (DPOAE)

To test the function of outer hair cells we measured DPOAEs as previously described [24], using the computerized National Hearing Instruments conducted in a sound-attenuating room at the Auditory Core for Department of Otolaryngology, Stanford University. DPOAE thresholds were calculated by interpolating the data and identifying when the signal was >−5 dB SPL and greater than two standard deviations above the noise floor. If no DPOAE response was detected even at our equipment limits of 80 dB SPL, we arbitrarily defined the threshold to be 80 dB.

### Immunofluorescence

The cochleae were dissected and perfused with 4% paraformaldehyde in PBS and were left in fixative solution for an additional 10 minutes in case of RIBEYE and SHANK1 staining, or for 1 h in case of staining for MYOVIIa, TUJ1, SYNAPTOPHYSIN, or PRESTIN. Samples were washed in PBS for 10 minutes and then blocked in 0.5% Triton X-100 plus 5% bovine serum albumin in PBS for 30 minutes at room temperature. Same blocking buffer was used for diluting antibodies. Primary antibodies were incubated at 4°C for 36 hours with light shaking on orbital shaker followed by three washes in 0.1% PBS-Tween with a final wash in PBS. Secondary antibodies were added for 1 hour at room temperature, followed by three washes in 0.1% PBS-Tween and PBS. The following antibodies were used primary - goat anti-CtBP2 (1∶200, Santa Cruz Biotechnology), rabbit anti-SHANK1 (1∶200, Neuromics), rabbit anti-MYOVIIa (1∶300, Proteus), mouse anti-TUJ1 (1∶200, Neuromics), anti-SYNAPTOPHYSIN (1∶200, Synaptic System), anti-PRESTIN (1∶300, clone N-20, Santa Cruz Biotechnology), and secondary – Alexa flour 488-conjugated anti-goat, Alexa flour 546-conjugated anti-rabbit, Alexa flour 488-conjugated anti-mouse (1∶500, Invitrogen). Cochleae were decalcified and in case of RIBEYE and SHANK1 markers further fixed in 4% paraformaldehyde in PBS for 15 minutes. Cochleae were washed in PBS and dissected into 2–3 pieces and mounted on microscopic slides in ProLong (Invitrogen) anti-fading media.

### Confocal Analysis of Synapse and Fiber Number

Images were collected on a Zeiss AxioVert confocal inverted microscope. For RIBEYE or SHANK1 analysis, cochlear frequency map [25] was estimated for every sample in order to localize hair cells from different frequency regions. Cochlear z-stacks from a selected frequency region were taken for each sample. Each stack contained the entire synaptic pole of 15–20 inner hair cells. Synapses were automatically counted through a series of optical sections using the Volocity 3D Image Analysis Software. The number of fibers crossing the tunnel of Corti was counted by hand per 500 um area using Z-stack images using Volocity 3D Image Analysis Software.

### RNA Extraction and Quantitative RT-PCR (qPCR)

RNA was extracted from whole cochlea dissected from wildtype mice at the postnatal day(s): P0 (n = 5), P6 (n = 3), P10 (n = 4), P15 (n = 3) and P29 (n = 4) using an Ambion RNAqueous Micro Kit (Thermo Fisher Scientific Inc., Waltham, MA) according to the protocol of the manufacturer. Extracted RNA was converted to cDNA using a High Capacity RNA to cDNA kit from Applied Biosystems (Foster City, CA). cDNA was further used in PCR assays with Taqman (Applied Biosystems) proprietary probes and primers for *C1qa* (assay ID Mm00432142_m1) and *H2-K1* (assay ID Mm01612247_m1). *GAPDH* (assay ID Mm99999915_g1) was used as the internal standard. All Taqman qPCR assays were performed on a BIO-RAD CFX96™ Real-Time System (Bio-Rad, Hercules, CA) with accompanying software for data analysis. Expression of the gene of interest was normalized with respect to the internal standard, *GAPDH*. Expression levels at various ages tested are shown relative to expression at P0. Error bars represent standard error of the mean (SEM).

### Statistical Analysis

Analysis of variance (ANOVA) was performed to determine significant differences between control and test groups. Following the ANOVA, planned comparisons were performed using two-tailed Student’s t-test. Values represent mean ± SEM, standard error of the mean, or ±SD, standard deviation, as specified in figure legends. Statistical analyses were conducted with SSPS software (IBM, Dublin, Ireland), Microsoft Excel (Microsoft Corp., Redmond, WA) or with Prism 6 (GraphPad Software Inc., La Jolla, CA). *p*<0.05 was considered to be statistically significant.

## Results

### Cochlear Afferent Synaptic Refinement is not Altered in *H2-K^b^d^b^* Double Knockout Mice

**Figure 1:**
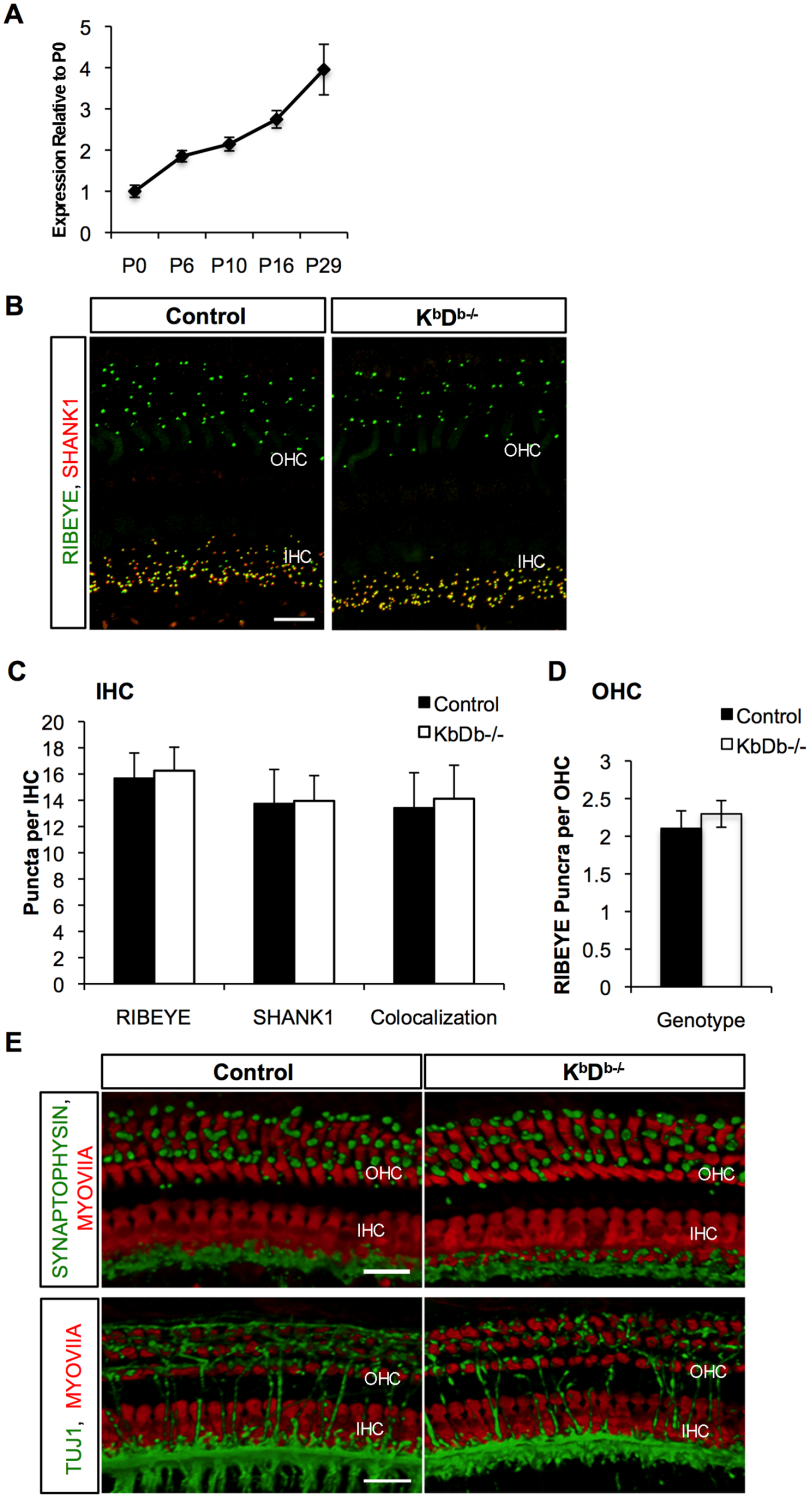
*K^b^D^b−/−^* mice have normal cochlear synaptic refinement. A) Whole cochlear tissue was isolated from mice during ages of synaptic refinement and maturation and *H2-K^b^* mRNA expression was analyzed. n = 3–5 mice per age group. Error bars show standard error of the mean. B) Double labeling fluorescent images showing RIBEYE (green) and SHANK1 (red) puncta to mark presynaptic and postsynaptic ribbons, respectively in mice P29 of age in both Control and *K^b^D^b−/−^* mice. Scale bar 10 um. C) and D) Quantification of RIBEYE and/or SHANK1 puncta beneath IHCs and OHCs of Control wild type and *K^b^D^b−/−^* mice, respectively, showed no significant differences between the two groups. *p* = 0.46 for RIBEYE, *p* = 0.9 for SHANK, and *p* = 0.70 for colocalized synaptic puncta in IHCs and *p* = 0.06 for RIBEYE in OHCs). E) Double labeling fluorescent images showing SYNAPTOPHYSIN (green) to mark efferent synapses or TUJ1 (green) for efferent and afferent fibers and MYOVIIa (red) to mark IHCs and OHCs in Control and *K^b^D^b−/−^* mice at P29 of age. Scale bar 10 um.

The age of onset of hearing in mice is around postnatal day 12 (P12), with synaptic refinement and functional maturation occurring during the first two postnatal weeks. Recent data demonstrates that *H2-K^b^* and *H2-D^b^* mRNAs are upregulated in the spiral ganglion at known times of synaptic refinement [19]. Our qPCR studies demonstrate that whole cochlear *H2-K^b^* mRNA expression increases from P0 to P29, as well as between P0 and P10 during the ages of synaptic refinement and maturation (Figure 1A). Because the knockdown of MHCI expression on the membrane of neurons results in a dramatic increase in synapse number in the cerebellum [14] we sought to determine if *K^b^D^b−/−^* double knockout mice have immature cochlear synaptic morphology, or delays in refinement. We therefore analyzed afferent and efferent fiber and synaptic patterning at P15 (data not shown) and at P29. We focused on the 16 kHz area of the cochlea, which has the highest afferent synaptic density and hearing sensitivity in mice [26], [27]. Pre- and post- synaptic puncta in the hair cells were visualized with immunostaining for afferent synapse markers RIBEYE/CtBP2 (presynaptic) and SHANK1 (postsynaptic). Quantification of pre- and post-synaptic puncta in both IHCs, displaying many afferent synapses, and OHCs, which have much less afferent synapses at P29 than IHCs, demonstrated no significant difference between *K^b^D^b−/−^* and control mice (Figure 1B–D). In addition, we observed no significant difference in the colocalization of RIBEYE and SHANK1 in IHCs of control and *K^b^D^b−/−^* animals: 15.7±1.9 RIBEYE puncta per IHC, 13.8±2.6 SHANK1 puncta per IHC (with 13.4±2.7 puncta colocalized) and 16.3±1.8 RIBEYE puncta per IHC, 14.0±1.9 SHANK1 puncta per IHC (with 14.1±2.6 puncta colocalized), respectively, which are expected values in an adult animal undergoing normal synaptic refinement [28]. SHANK1 immunofluorescence was absent in the OHCs at P29 in either group, which is consistent with the mature synaptic patterning. Consistently, there was no difference in RIBEYE staining in the OHCs between the *K^b^D^b−/−^* and control adult mice (2.1±0.23 puncta per OHC cell vs. 2.3±0.20 puncta per OHC; respectively). Further, SYNAPTOPHYSIN staining for efferent synapses was not altered in *K^b^D^b−/−^* mice in patterning or localization on the basal part of OHCs, suggesting proper efferent synapse formation (Figure 1E, Figure S1 in File S1). If proper refinement of the synapses in the OHCs did not occur, we would expect more fibers crossing the tunnel of Corti and innervating the OHCs. TUJ1 immunostaining for efferent and afferent fibers’ synapses indicated that innervation patterns were grossly normal in mutants compared to controls (Figure 1E). The data presented above suggest that the number and structure of efferent and afferent fibers and synapses are comparable in the *K^b^D^b−/−^* mice and controls.

### *C1qa^−/−^* Mice do not Have a Disruption in Cochlear Synaptic Refinement

**Figure 2:**
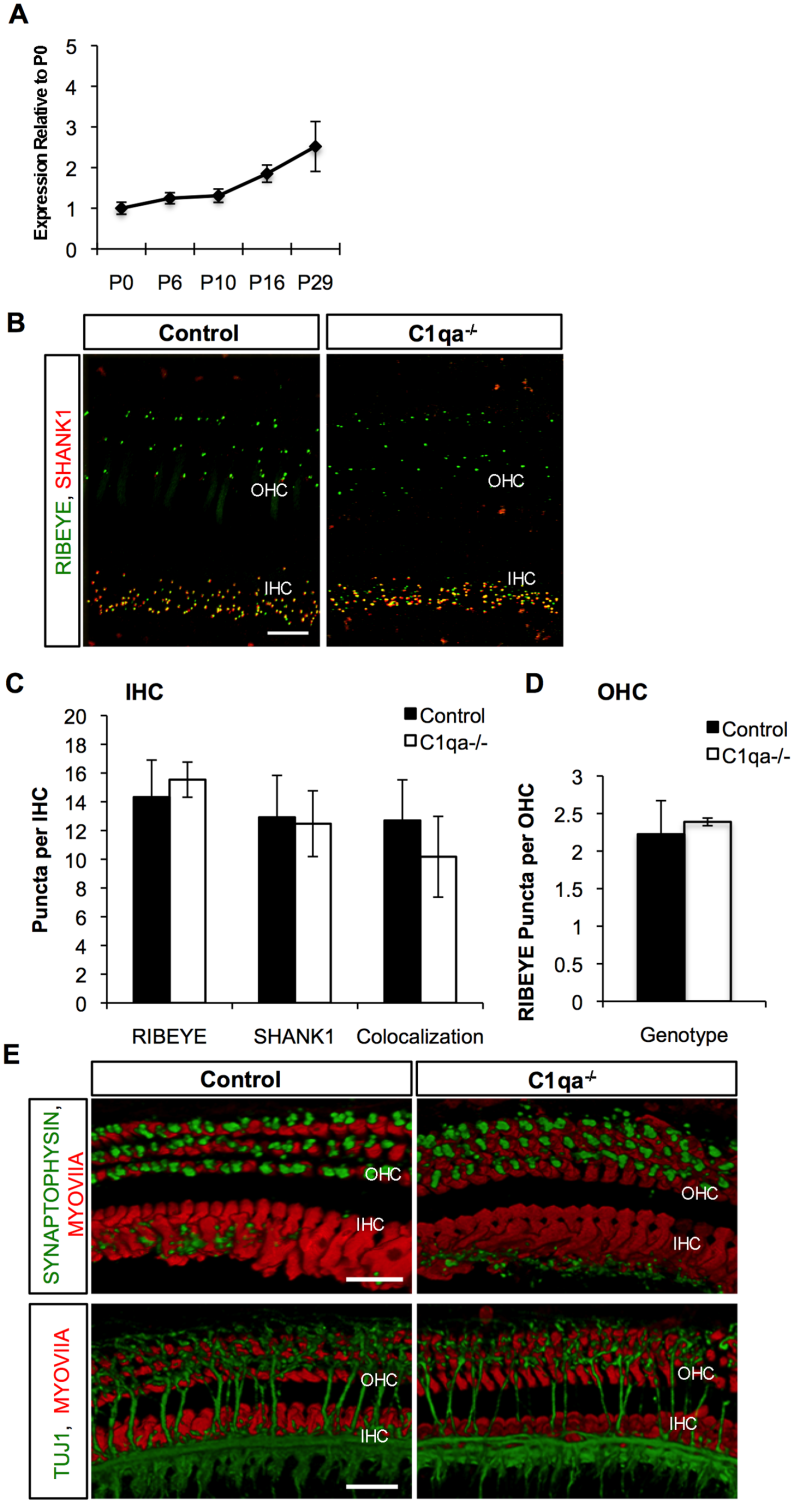
*C1qa^−/−^* mice have no alteration in synaptic refinement. A) Whole cochlea tissue was isolated from mice during ages of synaptic refinement and maturation and *C1qa* mRNA expression was analyzed. n = 3–5 mice per age group. Error bars show standard error of the mean. B) Double labeling fluorescent images showing RIBEYE (green) and SHANK1 (red) puncta to mark presynaptic and postsynaptic ribbons, respectively in mice P29 of age in both Control and *C1qa^−/−^* mice. Scale bar 10 um. C) and D) Quantification of RIBEYE and/or SHANK1 puncta beneath IHCs and OHCs of Control wild type and *C1qa^−/−^* mice, respectively, showed no significant differences between the two groups. *p* = 0.27 for RIBEYE, *p* = 0.8 for SHANK, and *p* = 0.22 for colocalized synaptic puncta in IHCs and *p* = 0.517 for RIBEYE in OHCs. E) Double labeling fluorescent images showing SYNPATOPHYSIN (green) to mark efferent synapses or TUJ1 (green) for efferent and afferent fibers and MYOVIIa (red) to mark IHCs and OHCs in Control and *C1qa^−/−^* mice at P29 of age. Scale bar 10 um.

qRT-PCR demonstrated that *C1qa* mRNA expression in the normal mouse cochlea increased in the first two postnatal weeks and continued to increase past this age (Figure 2A). Because *C1qa^−/−^* mice exhibit large sustained defects in CNS synapse elimination in the retina and in the cerebral cortex, we quantitatively and qualitatively analyzed cochlear synapses at P29 in order to detect differences in the mutants with respect to wild type controls. Quantification of pre- and post-synaptic puncta beneath the IHCs, with markers RIBEYE and SHANK1 respectively, did not show significant differences between *C1qa^−/−^* and control mice (Figure 2B–D). We next quantified the colocalization of RIBEYE and SHANK1 in these mice as a reflection of synapse formation and function. We observed 12.7±2.8 colocalized RIBEYE and SHANK1 puncta per IHC in control mice and 10.18±2.8 colocalized RIBEYE and SHANK1 puncta per IHC in *C1qa^−/−^* animals. The number of observed puncta in our adult control animals was similar to expected values in an adult animal undergoing normal synaptic refinement [28]. The *C1qa^−/−^* mice had a slight but non-significant decrease in the level of colocalization compared to controls, which is attributable to high variability and a slight increase in number of RIBEYE puncta (not significant when compared to controls; 14.3±3.58 puncta per IHC in controls vs. 15.5±1.2 puncta per IHC in mutants). Because IHC refinement and OHC refinement might be regulated by different mechanisms, we next analyzed OHC refinement in *C1qa^−/−^* compared to control mice. We did not observe SHANK1 expression in the OHC at P29 in either group, consistent with normal synaptic patterning. There was no difference in RIBEYE staining in the OHCs between and control and *C1qa^−/−^* adult mice (2.2±0.45 and 2.4±0.05 puncta per OHC respectively). Efferent synapse formation, as determined by SYNAPTOPHYSIN staining in the basal part of the OHCs, was also comparable in *C1qa^−/−^* and controls (Figure 2E). Efferent and afferent fibers crossing the tunnel of Corti, as determined by TUJ1 staining, were also not obviously different in *C1qa^−/−^* versus controls (Figure 2E). In summary, our data indicate that the efferent and afferent fiber patterning in *C1qa^−/−^* animals are not detectably different from controls. Similar to *H2-K^b^D^b^* genes, *C1qa* expression does not appear to be necessary for proper afferent and/or efferent synapse formation and synaptic refinement, based on the parameters measured, occurs normally in mutant mice.

### Knockout of MHC Class I Genes Causes Hearing Impairment

**Figure 3:**
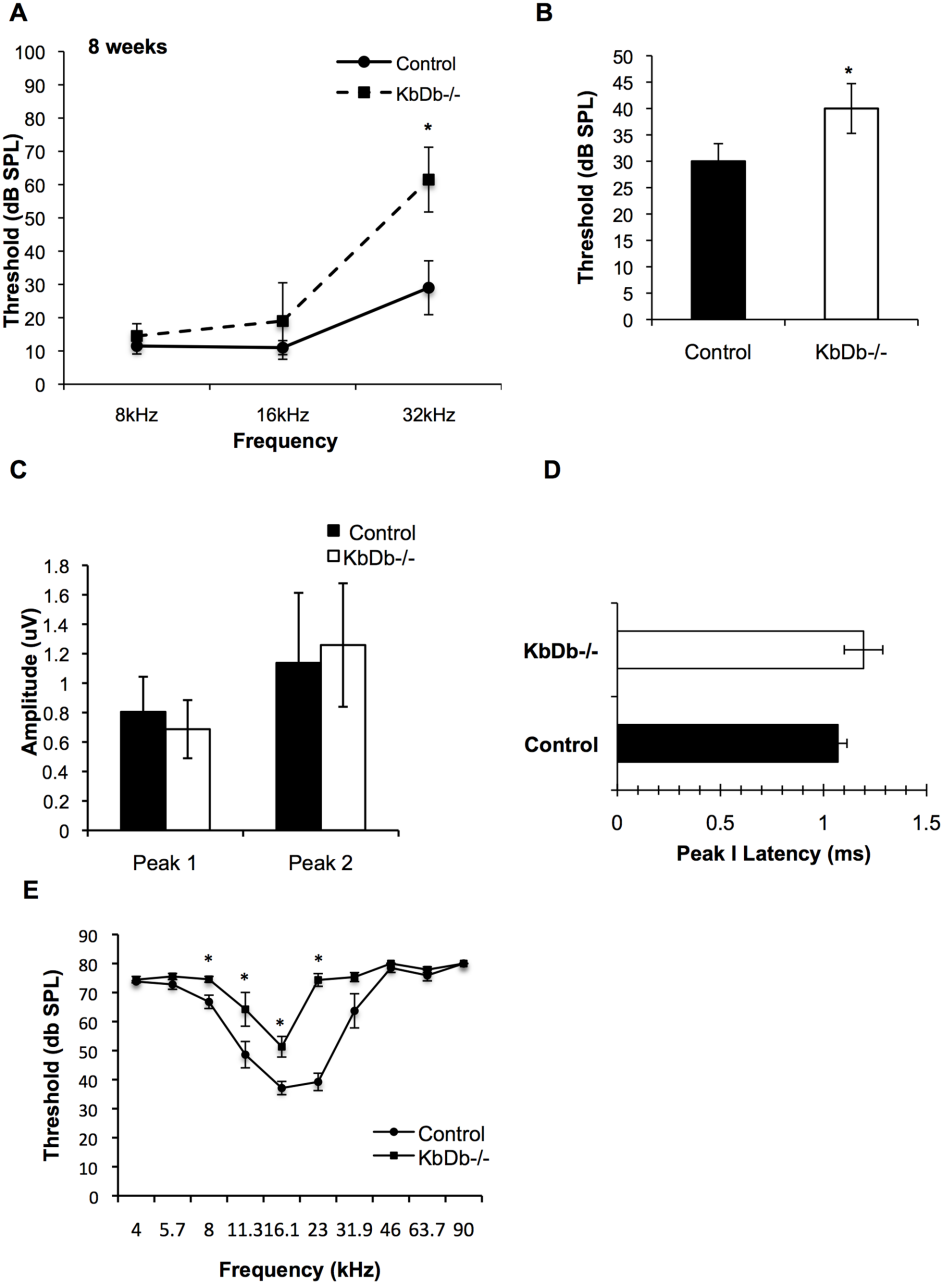
ABR and DPOAE thresholds are increased in *K^b^D^b−/−^* mice. Average ABR (A–B) and DPOAE (E) thresholds are shown for mice at 8-weeks of age. Significantly increased ABR thresholds were recorded in *K^b^D^b−/−^* mice with pure-tone 32 kHz stimuli (A) and with broad-band clicks (B). *p = 2.5E-07 and *p* = 0.0001 for 32 kHz and broad-band clicks, respectively. ABR waveform amplitude of peaks 1 and 2 (C) and latency analysis of peak 1 (D) measured at the 16 kHz frequency stimulus and at 40 dB demonstrated no significant differences between Control and *K^b^D^b−/−^* cohorts. *p* = 0.44, *p* = 0.67 and *p* = 0.57 for peak 1 and peak 2 amplitudes and peak 1 latency, respectively. Increased DPOAE thresholds were recorded at 8, 11.3, 16.1, and 23 kHz frequencies (E). n = 10 (Control) and 9 (*K^b^D^b−/−^*) mice per group. *p = 0.007, *p* = 0.04, *p* = 0.003 and *p* = 1.25E-07 for 8, 11.3, 16 and 23 kHz, respectively. Error bars show SDs in (A), (B), (C), (D), and SEMs in (E). *p<0.05 for (E).

**Figure 4:**
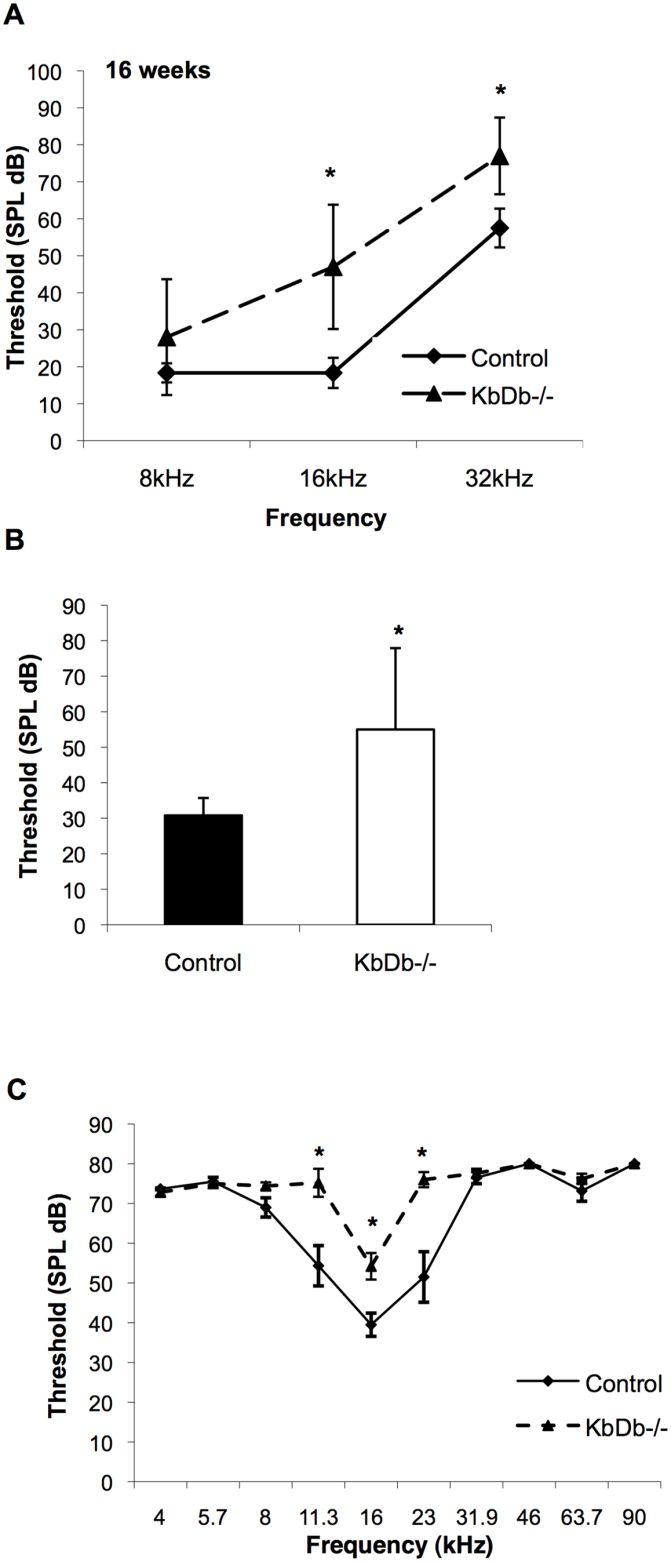
Average ABR and DPOAE thresholds continue to elevate with age in *K^b^D^b−/−^* mice. Average ABR (A–B) and DPOAE (C) thresholds are shown for mice at 16-weeks of age. Significantly increased ABR thresholds were recorded in *K^b^D^b−/−^* mice with pure-tone 16, and 32 kHz stimuli (A) and with broad-band clicks (B). *p = 0.017, *p* = 0.001 and *p* = 0.032 for 16 kHz, 32 kHz and broad-band clicks, respectively. Increased DPOAE thresholds were recorded at 11.3, 16, and 23 kHz frequencies. *p = 0.006, *p* = 0.005 and *p* = 0.005 for 11.3, 16 and 23 kHz, respectively. n = 6 (Control) and 5 (*K^b^D^b−/−^*) mice per group (C). Error bars show SDs in (A), (B) and SEMs in (C).

Although our morphological data demonstrate normal synaptic refinement and development in the cochlea of mice lacking both *H2-K^b^* and *H2-D^b^* genes (*K^b^D^b−/−^*), whether MHC class I genes in play a role in auditory function is unknown. We analyzed auditory brain stem responses (ABRs) of *K^b^D^b−/−^* mice at 8 weeks of age and found significant hearing impairment as demonstrated by an increase in frequency threshold compared to age/sex-matched C57BL/6 controls (Figure 3A–B, Figure S2 in File S1). A significant increase in thresholds was seen for click and 32 kHz stimuli. It has been shown that the ABR threshold does not always reflect subtle differences in the number of neurons that are firing [29]–[31]. Therefore, we decided to study the wave amplitude and latency to reveal differences in the average of all active synapses and firing neurons in control versus mutant animals. We measured the peak amplitude of wave 1, and also the time elapsed from the onset of stimulus to peak 1 (response latency). There were no differences in peak 1 amplitudes or peak 1 latencies between the two cohorts that would be suggestive of a possible deficiency in neural firing (Figure 3C–D) [29]–[31]. Previous work suggests that the complement cascade is involved in axonal but not dendrite synapse pruning in the CNS [16]. We assumed that a lack of refinement of axonal synapses would result in the retention of functional synapses, and this would lead to an increase in the number of firing synapses. Therefore, we analyzed the second wave amplitude (peak 2) as an estimate of the number of axonal synapses firing. Peak 2 amplitudes were also comparable between the *K^b^D^b−/−^* mice and controls (Figure 3C). To determine whether the difference in average ABR thresholds at the higher frequency of 32 kHz is caused by alterations of OHC function, we tested the distortion-product otoacoustic emission (DPOAE) in these mice. We observed significant differences in the mutants as compared to controls at 8 kHz, 11.3 kHz, 16.1 kHz, and 23 kHz (Figure 3E). We also analyzed the hearing impairment phenotype at 16 weeks of age. Although the control mice had elevated thresholds due to their C57BL/6 background displaying early onset age-related hearing loss [32], we observed additional threshold elevations in both ABR and DPOAE thresholds in the *K^b^D^b−/−^* mice (Figure 4A–C). Together, these data demonstrate that MHC class I genes are necessary for normal hearing when tested in adult mice of 8 and 16 weeks of age. To exclude other potential factors that might contribute to this phenotype, we examined the knockout mice for signs of inflammation in the middle and inner ear, suggesting an absence of middle ear infection. Further, the hearing impairment was not accompanied by gross morphological alterations in the cochlear duct as determined by examination of plastic-embedded sections (data not shown). However, we cannot rule out the possibility that elevated thresholds in the mutant mice are due to hearing loss involving conductive structures in the middle ear. Also, immunostaining with antibodies to PRESTIN, the motor protein expressed in outer hair cells, showed no apparent difference in morphology of outer hair cells in adult *K^b^D^b−/−^* mice compared to controls (Figure S3 in File S1). Further investigations are needed to understand the hearing impairment caused by mutations in in these genes.

### *C1qa* Expression is not Necessary for Proper Hearing

**Figure 5:**
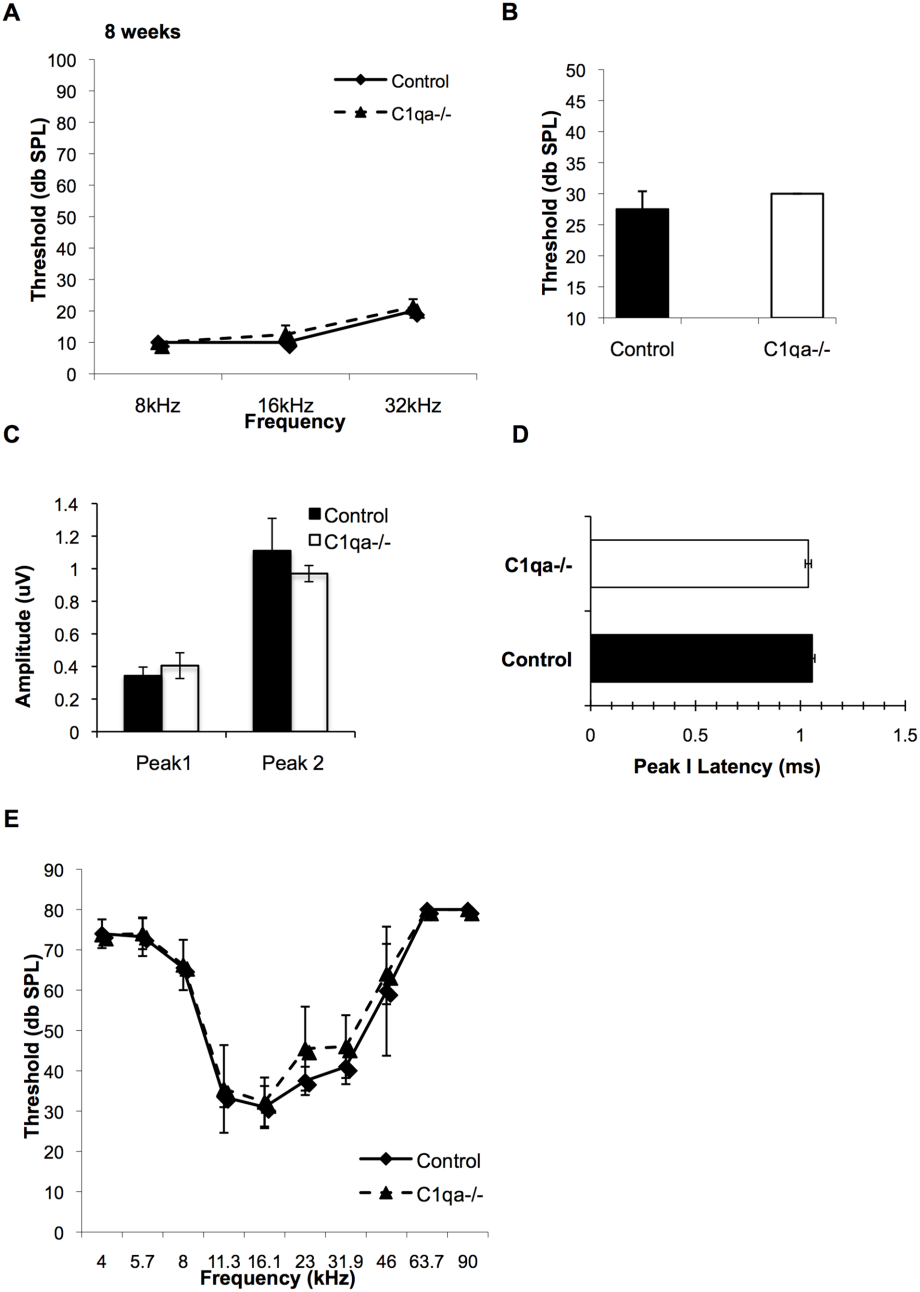
ABR and DPOAE thresholds are not altered in *C1qa^−/−^* mice. Average ABR (A–B) and DPOAE (D) thresholds are shown for mice at 8-weeks of age. Student’s t-test did not reveal significant differences in ABR thresholds that were recorded in *C1qa^−/−^* mice with pure-tone 8, 16, and 32 kHz stimuli (A) or broad-band clicks (B) compared to controls. ABR waveform amplitude of peaks 1 and 2 (C) and latency analysis of peak 1 (D) measured at the 16 kHz frequency stimulus and at 40 dB demonstrated no significant differences between Control and *C1qa^−/−^* cohorts. There were no differences in DPOAE thresholds at any frequency tested between the cohorts (E). *p*>0.05 for all comparisons. n = 4 mice per group. Error bars show SDs.

We next sought to determine the role of the complement cascade in promoting proper hearing in the mouse cochlea. The ABR thresholds of 8-week-old *C1qa^−/−^* mice were compared to that of C1qa expressing control mice (Figure 5A–B). There was no difference in hearing thresholds at the 8, 16, and 32 kHz frequencies as well as in response to broadband click stimuli measured between the two genotypes. We analyzed the ABR data at 16 kHz, within the most sensitive frequency range for mice for peak 1 amplitudes and latencies. We did not observe a shift in peak 1 amplitude or latency in *C1qa^−/−^* mice compared to controls (Figure 5C–D). Since peripheral projections in the cochlea are considered long dendrites and may be C1qa independent, we analyzed the second wave amplitude (Peak 2) as an estimate of the number of axonal synapses firing and observed that it is also unaffected in *C1qa^−/−^* mice (Figure 5C). In addition, no difference in OHC function was revealed by DPOAE measurements between *C1qa^−/−^* and control mice (Figure 5E).

### Lack of *C1qa* Expression is not Protective in Progressive Hearing Loss

**Figure 6:**
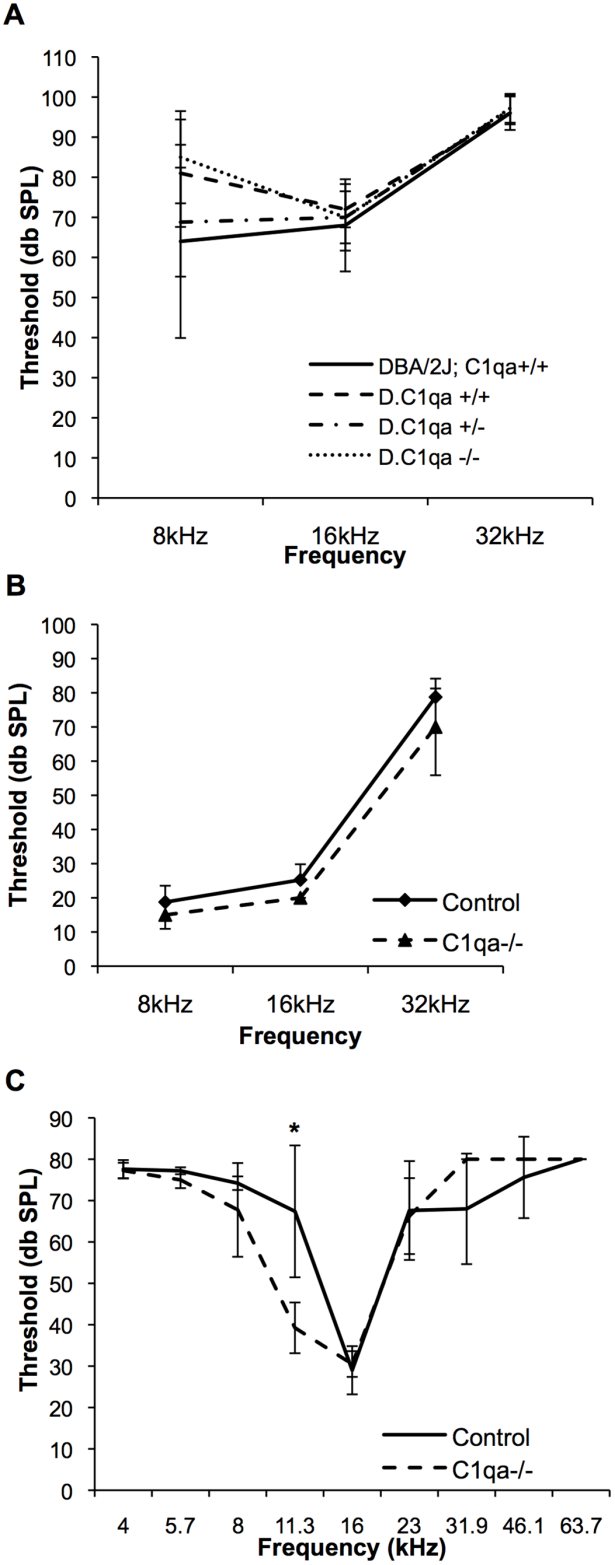
*C1qa^−/−^* mice are not protected from progressive or age-related hearing loss. A) Average ABR thresholds for *DBA/2J-C1qa^+/+^* controls (n = 5) and congenic *D2.B6-C1qa^+/+^* (n = 5), *D2.B6-C1qa^+/−^* (n = 8), and *D2.B6-C1qa^−/−^* (n = 9) mice at 1-month of age are shown. Average ABR (B) and DPOAE (C) thresholds are shown for *B6-C1qa^−/−^* (n = 4) and B6 control (n = 5) mice at 10-months of age. *(*p* = 0.01). Error bars show SDs.

We next determined if a lack of *C1qa* expression would protect mice from progressive or age-related hearing loss (AHL) in two different mouse models. Mice from the DBA/2J strain suffer from early-onset, progressive hearing loss [33], [34]. All of the genetic components contributing to the hearing loss phenotypes in DBA/2J mice have not been accounted for as of yet. The DBA/2J strain is also a well-known mouse model of glaucoma [35]. Retinal ganglion cell loss and degeneration of the optic nerve are characteristics of this neurodegenerative disease model. In early stages of glaucoma in this model, C1qa protein was re-expressed and localized to adult retinal synapses [16]. Further, *C1qa* deficiency on the DBA/2J background prevented damage to the retina and optic nerve and protected mice from glaucoma [23]. We hypothesized that the hearing loss observed in the DBA/2J mice is related to *C1qa*-regulated spiral ganglion cell loss. To test this hypothesis, we analyzed congenic mice that are deficient for *C1qa* on the DBA/2J mouse background (*D2.B6-C1qa^−/−^*). ABR thresholds for DBA/2J mice with wild-type expression of *C1qa* (*DBA/2J-C1qa^+/+^*) are shown in Figure 6C. We compared the one-month thresholds of the progeny from an intercross of *DBA/2J-C1qa^+/+^* and *D2.B6-C1qa^−/−^* F1 hybrids resulting in F2 progeny with *C1qa^+/+^*, *C1qa^+/−^*, and *C1qa^−/−^* genotypes all on a DBA/2J background. However, lack of *C1qa* failed to protect these mice from hearing loss (Figure 6A). C57/BL6 mice have an AHL mutation of *Cadherin 23* causing OHC degeneration and increasing the susceptibility to age-related hearing loss [32]. It has recently been shown that mice deficient in *C1qa* have less cognitive and memory loss with age compared to their littermate controls [18]. We hypothesized that *C1qa* synaptic re-expression in aging might mediate AHL and that a lack of *C1qa* expression in our *C1qa^−/−^* mice might protect against age-related hearing loss and OHC death. ABR thresholds of mice at 10 months of age were not different compared to control mice (Figure 6B), demonstrating the absence of a protective effect. A small reduction in DPOAE thresholds was seen at 11.3 kHz in mice lacking *C1qa* expression at 10 months of age but not at other frequencies (Figure 6C). In summary, these data suggest that although MHCI genes may be necessary for proper hearing function and a lack of *C1qa* in the brain exerts a protective effect in the central nervous system, its absence in the cochlea does not protect from AHL.

## Discussion

The cochlea is a good model system to study innervation and synaptic refinement because it contains only a few cell types that undergo a well-orchestrated developmental innervation and postnatal refinement program [28], [36]–[40]. Microarray gene expression analysis by other groups has revealed a postnatal upregulation of MHC class I, MHC class II, and complement pathway genes in spiral ganglion neurons [19]. We extended the existing microarray studies with quantitative expression analyses over the course of postnatal refinement and found that *H2-K^b^* and complement component *C1qa* mRNA is increasingly expressed in the mouse cochlea during the first four postnatal weeks. The fact that MHCI and complement genes have recently been implicated in synaptic refinement such as synaptic elimination in development and aging [13]–[18] spurred our interest in investigating a potential role for these genes in the postnatal cochlea.

Interestingly, a lack of major histocompatibility genes *H2-K^b^* and *H2-D^b^*, but not complement component *C1qa*, leads to an increase in hearing threshold at high frequency. This result leads to the question of whether sustained down-regulation of immune gene expression, leads to defective clearance of dead and dying cells that could become antigenic and drive organ-specific autoimmunity [41], [42]. Assuming that the primary role of cochlear glial-like supporting cells, like that of other glial cells, is to support neuronal function, the challenge now is to determine what sort of pathological scenarios, like noise exposure or aging, could transform these supporting cells into auto aggressive effector cells that attack healthy neurons and cause neurodegeneration. In the CNS, glial cells are capable of performing both neuroprotective and neurodestructive functions [43]. Under physiological conditions, glial cells exhibit a deactivated phenotype that is associated with the production of anti-inflammatory and neurotrophic factors [44]. Microglia switch to an activated phenotype in response to pathogen invasion or tissue damage and thereby promote an inflammatory response that serves to further engage the immune system and initiate tissue repair. Glial cell dysfunction may become manifest in a number of ways, including a decreased ability to clear dead cells, decreased ability to produce neurotrophic factors, as well as increased neurotoxicity and synapse elimination. In our case, we hypothesized that *C1qa* knockout mice would lack the ability to clear out cellular debris caused by continuous noise exposure and this would lead to local autoimmunity in the cochlea. However, our aging ABR data suggests that this may not lead to hearing impairment. We also screened for *C1qa* playing a protective role in age-related hearing loss in the congenic *D2.B6-C1qa* model and found that a lack of *C1qa* does not prevent this condition. Because expression of MHCI proteins on the surface of cells may limit post-stress (post injury) synaptic loss and neuronal cell death, we might expect the *K^b^D^b−/−^* mice to lack this protective function compared to controls. Noise exposure and/or aging may reveal an important protective role for these molecules. Further studies will need to be done to determine if autoimmunity has arisen mediating the hearing loss associated with *H2-K^b^/H2-D^b^* deficiency.

Our study demonstrates that at an individual level these immune proteins are not necessary for proper synaptic refinement and lack of their expression is not sufficient to alter auditory circuit maturation. In gene families with many members, similar genes may functionally compensate for the deleted gene due to redundancy. Work ongoing in our laboratory aims to identify thyroid hormone-regulated gene profiles by microarray analysis in order to understand the genetic signature controlling auditory synaptic refinement and maturation at the spiral ganglion neuron level. We expect that along with MHC and complement pathways altered, we will also identify other genes involved in the “eat-me” signal of phagocytosis or clearance [45], [46]. It is possible that many of the molecules in “eat-me” signaling are involved in the cochlea and only by deleting the pathways will we see a phenotype. It is also possible that these immune system pathways are not playing the main role in eliminating a weakened synapse or neuron but have a secondary function (like cleaning out debris) that will be compensated for by different pathways that remain to be elucidated. This study highlights the challenge of identifying the molecular mechanisms of synapse removal and refinement necessary for proper cochlear development. We performed a candidate gene based approach because these genes were associated with processes shared by the cochlea, brain and retina. Our study revealed that the individual genes regulating these processes in other neural tissues are not shared or regulated in the same manner in the cochlea as in the CNS. Although increased expression of *H2-K^b^*, *H2-D^b^*, and *C1qa* genes coincides with ages of auditory synaptic refinement, their functions and roles in the developing cochlea remain unclear.

## Supporting Information

File S1 **Contains Figures S1–S3.**
(DOCX)
