# Supplementary material for: A Lack of Immune System Genes Causes Loss in High Frequency Hearing but Does Not Disrupt Cochlear Synapse Maturation in Mice
Source: PLoS One. 2014 May 7;9(5):e94549. doi: 10.1371/journal.pone.0094549 (PMC4012943; doi:10.1371/journal.pone.0094549)
Supplement: File S1 — Contains Figures S1–S3. (DOCX) [file pone.0094549.s001.docx]

***Supplemental Information***

**Figure S1: K^b^D^b-/-^ mice have normal cochlear synaptic refinement.** High magnification double labeling fluorescent images show SYNAPTOPHYSIN marking efferent synapses and MYOVIIa marking IHCs and OHCs in Control and K^b^D^b-/-^ mice at P29 of age. Scale bar 10um.


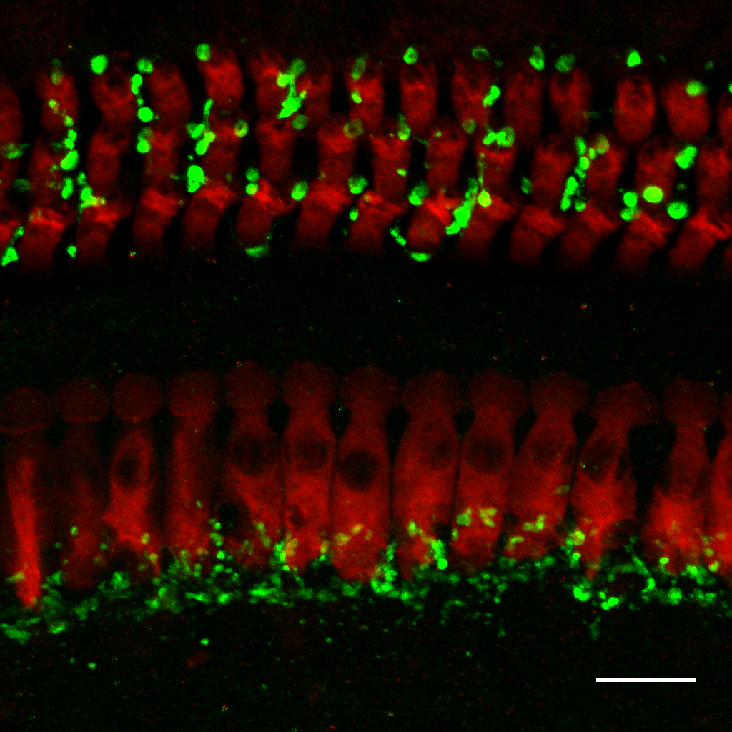

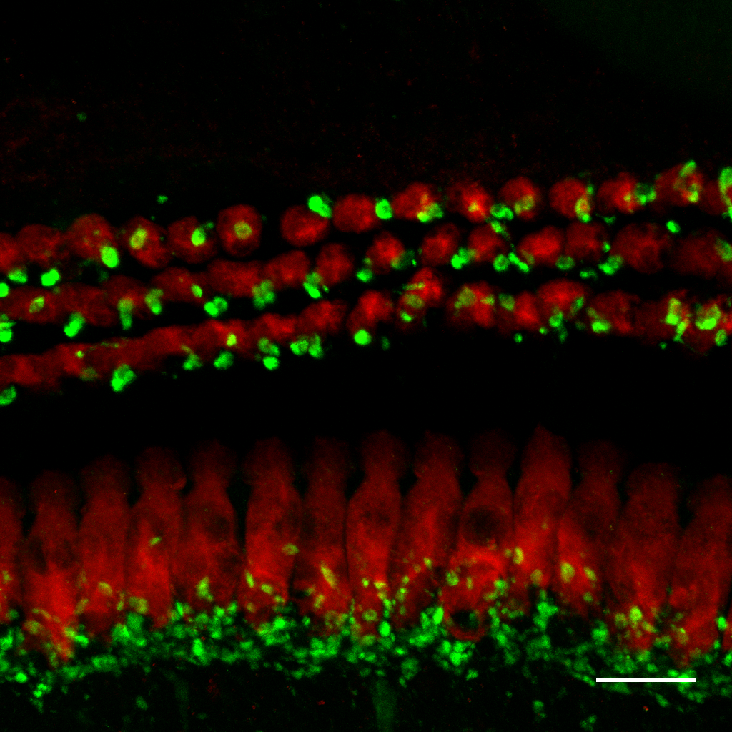


OHC

IHC

OHC

IHC

**Control**

**K^b^D^b-/-^**

**SYNAPTOPHYSIN + MYOVIIA**

**Figure S2: ABR and DPOAE thresholds are not altered in control K^b^D^b^ mice.** Average ABR (A) and DPOAE (B) thresholds are shown for mice at 4-weeks of age. No significant difference in ABR thresholds were recorded in Control wild-type K^b^D^b^ mice with pure-tone 8, 16, 32kHz stimuli (A) compared to Control wild-type C57BL/6J mice or in DPOAE thresholds at any of the frequencies tested (D). n=4 (control C57BL/6J) and n=7 (control K^b^D^b^) mice per group. Error bars show standard deviation.

**A) B)**

**Figure S3: K^b^D^b-/-^ mice do not have altered expression of PRESTIN.** Fluorescent images from the mid-turns of P29 mice cochleae with the OHC motor protein PRESTIN (red) indicate no alteration in K^b^D^b-/-^ mice compared to Controls. Scale bar 10um.
